# Urea-Mediated Cross-Presentation of Soluble Epstein-Barr Virus BZLF1 Protein

**DOI:** 10.1371/journal.ppat.1000198

**Published:** 2008-11-07

**Authors:** Sascha Barabas, Regina Gary, Tanja Bauer, Juha Lindner, Petra Lindner, Birgit Weinberger, Wolfgang Jilg, Hans Wolf, Ludwig Deml

**Affiliations:** 1 Institute of Medical Microbiology, University of Regensburg, Germany; 2 Department of Hematology and Oncology, University of Erlangen-Nuernberg, Germany; 3 Institute for Biomedical Aging Research, Austrian Academy of Sciences, Austria; University of Wisconsin-Madison, United States of America

## Abstract

Soluble extracellular proteins usually do not enter the endogenous human leukocyte antigen (HLA) I–dependent presentation pathway of antigen-presenting cells, strictly impeding their applicability for the re-stimulation of protein-specific CD8^+^ cytotoxic T lymphocytes (CTL). Here we present for the Epstein-Barr virus (EBV) BZLF1 a novel strategy that facilitates protein translocation into antigen-presenting cells by its solubilisation in high molar urea and subsequent pulsing of cells in presence of low molar urea. Stimulation of PBMC from HLA-matched EBV-seropositive individuals with urea-treated BZLF1 but not untreated BZLF1 induces an efficient reactivation of BZLF1-specific CTL. Urea-treated BZLF1 (uBZLF1) enters antigen-presenting cells in a temperature-dependent manner by clathrin-mediated endocytosis and is processed by the proteasome into peptides that are bound to nascent HLA I molecules. Dendritic cells and monocytes but also B cells can cross-present uBZLF1 *in vitro*. The strategy described here has potential for use in the development of improved technologies for the monitoring of protein-specific CTL.

## Author Summary

CD8^+^ T lymphocytes (CTL) play a key role in the immunological control of persistent intracellular pathogens and tumors. Thus, the development of improved technologies for the monitoring and expansion of protein-specific CTL represents a major challenge in clinical immunology. CTL specifically target infected cells through the recognition of peptides displayed by surface exposed HLA class I molecules. In most cell types, HLA class I–associated peptides are generally derived from cytosolic proteins. In contrast, delivery of soluble exogenous proteins to the endogenous HLA class I processing pathway is scarce. Here we exemplified with the Epstein-Barr virus immediate early protein BZLF1 a novel and simple urea-based strategy to deliver soluble proteins to the class I processing pathway of antigen-presenting cells by cross-presentation. We showed that urea formulated BZLF1 but not urea-free BZLF1 reveals a strong capacity to reactivate CD8^+^ T cells in blood cells of EBV-positive donors. Accordingly, dendritic cells, monocytes, but also B cells are able to cross-present BZLF1-derived epitopes to CTL. This technology could improve the development of T cell diagnostics for microbial diseases and may facilitate a novel strategy for the expansion of protein-specific CTL for therapeutic application.

## Introduction

Cytotoxic CD8^+^ T lymphocytes (CTL) play a key role in the immunological control of persistent intracellular pathogens and tumors. CTL specifically target infected cells through the recognition of peptides, which are displayed by surface exposed HLA class I molecules. In most cell types, HLA class I-associated peptides are generally derived from intracellularly synthesized proteins. For the processing in the endogenous antigen presentation pathway, peptides are generated by the proteasome and translocated by the transporter associated with antigen processing (TAP) to the lumen of the endoplasmic reticulum, where they are trimmed and loaded onto HLA class I molecules [1]–[3]. The resulting complexes of mature HLA class I molecules and peptides are transported through the secretory pathway for display on the cell surface, where they can interact with CTL. In contrast, exogenous soluble antigens are typically internalized by antigen-presenting cells (APC) via the endosomal route, proteolytically fragmented in acidic endosomal compartments and loaded onto HLA class II molecules to produce a mature complex, which is capable of interacting with CD4^+^ T helper (Th) cells. However, APC have the capacity to internalize pathogens as well as infected and abnormal cells from their environment and direct them to the endogenous HLA class I processing machinery by a process termed cross-presentation. Efficient cross-presentation of exogenous antigens is achieved by dendritic cells and macrophages [4]–[6], but also by B cells [7], endothelial cells [8] and neutrophils [9] via various routes for antigen delivery from endosomes and phagosomes to the cytosol [10],[11] or by direct processing of captured antigens in intracellular vesicles or at the plasma membrane [10],[12],[13]. Yet, evidence for the stimulation of CTL by soluble exogenous proteins is scarce.

The development of protein delivery systems for APC is one of the main challenges in T cell diagnostics, vaccinology and cellular therapy. Current strategies include intracellular synthesis of foreign antigens by infection of APC with microbial live vectors or transfection with expression vectors and RNA [14],[15]. In addition, coupling of antigens to micro beads [16], antibodies [17],[18], lipids [19], heat shock proteins [20] and arginine-rich protein transduction domains (PTD) [21] confers delivery to the HLA class I processing machinery. However, a variety of practical and theoretical concerns, such as restrictions in the translocation of individual antigens, the lack of biological inertness of the transfer vectors and/or difficulties with the preparation and use of more complex experimental systems preclude versatile applications in diagnostics, therapy and prevention of infectious diseases and tumors.

Here we demonstrate a substantial improvement of HLA class I-dependent antigen presentation to CTL by urea treatment, which facilitates an efficient antigen translocation to the endogenous cytosolic processing pathway.

## Results

### Urea-adjuvated soluble EBV BZLF1 specifically stimulates CTL

Soluble extracellular proteins are almost exclusively processed by the exogenous pathway for presentation on HLA class II molecules, thus strictly limiting their capacity to activate specific CTL responses [22],[23]. Here we investigated, whether the denaturation of protein in a high molar urea solution and its subsequent incubation with APC in the presence of low concentrations of urea mediates an improved protein delivery into the endogenous antigen processing and presentation pathway.

**Figure 1:**
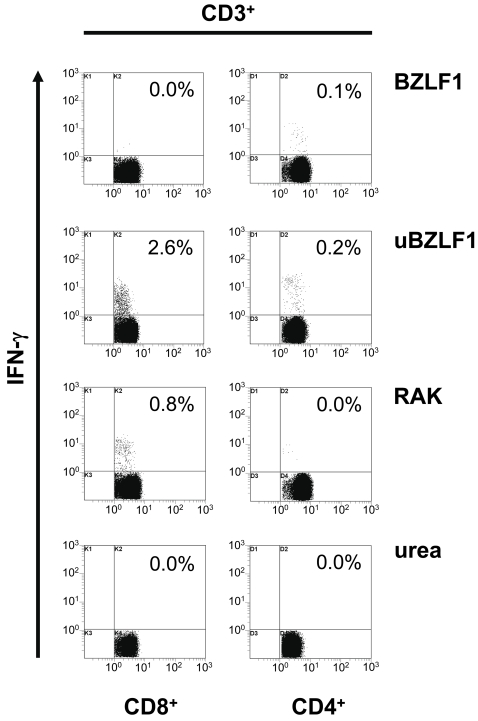
Urea-treated BZLF1 (uBZLF1) but not untreated BZLF1 restimulates specific CD8^+^ cytotoxic T lymphocytes. Whole blood from an EBV-seropositive, HLA B8-positive donor was stimulated with either 10 µg/ml of BZLF1 or uBZLF1 and as controls with 10 µg/ml of RAK peptide and 0.04 M urea. Cells were incubated at 37 C° for 7 h with 10 µg/ml BFA added for the last 4 h. Shown is the percentage of CD3^+^CD8^+^ or CD3^+^CD4^+^ cells expressing IFN-γ from a representative stimulation of eight independent experiments using a total of 4 different donors. Plots show log fluorescence intensity.

**Figure 2:**
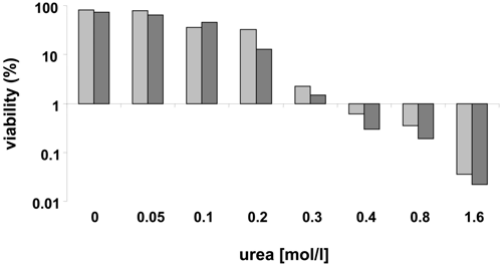
Residual urea in uBZLF1 pulsed cell cultures reveals no significant cytotoxic effect on lymphocytes. PBMC of two healthy adult individuals, indicated by the light and dark grey bars, were incubated with increasing concentrations of urea. Cell viability was determined after 20 h by propidium iodide (PI) staining.

As a model antigen we used the EBV BZLF1 protein, since it has been previously described to harbor an immunodominant B8-restricted CTL epitope termed RAK [24], thus offering a suitable read-out system for the detection of BZLF-specific CTL reponses. Heparinized whole blood obtained from HLA B8-positive EBV-positive individuals was stimulated with either 10 µg/ml urea-adjuvated BZLF1 (uBZLF1) or dialyzed urea-free BZLF1, while the synthetic RAK peptide and a 0.04 M urea solution served as positive and negative controls, respectively. Exposure of blood cells with uBZLF1 induced the reactivation of substantial numbers of CTL as shown by intracellular IFN-γ staining, which was not seen in cells stimulated with BZLF1 or 0.04 M urea (Fig. 1), even after prolongued incubation times (data not shown). In addition, the frequencies and ratios of specifically reactivated CTL upon stimulation with uBZLF1 and the RAK peptide differed significantly in blood samples of individual donors. The removal of urea from uBZLF1 by extensive dialysis resulted in an almost complete loss of its capacity to stimulated CD8^+^ T cells. Furthermore, we observed only low or undetectable numbers of IFN-γ producing CD4^+^ Th cells in response to stimulation with uBZLF1 (Fig. S1). Comparable results were achieved using recombinant cytomegalovirus (CMV) pp65 protein (Miltenyi Biotec). Urea-formulated pp65 (upp65) but not urea-free dialyzed pp65 induced significant reactivation of pp65-specific CTL, whereas removal of urea did not significantly influence the capacity of (u)pp65 to restimulate CD4^+^ T cells (Fig. S2). Dose titration studies with urea revealed no significant cytotoxic effects at concentrations of 0.04 M urea present in uBZLF1-stimulated peripheral blood mononuclear cells (PBMC) (Fig. 2). Beyond that, the applied urea solution did neither induce the secretion of cytokines from PBMC (Fig. S3) nor activate immature dendritic cells for the upregulation of costimulatory (CD80, CD83 and CD86) and MHC class-II molecules (Fig. S4). These data indicate that urea formulation mediates an efficient transfer of EBV BZLF1 into the endogenous HLA class I presentation pathway.

### Transfer of uBZLF1 to the HLA class I presentation pathway is not mediated by a PTD-like domain within the C-terminal region of BZLF1

**Figure 3:**
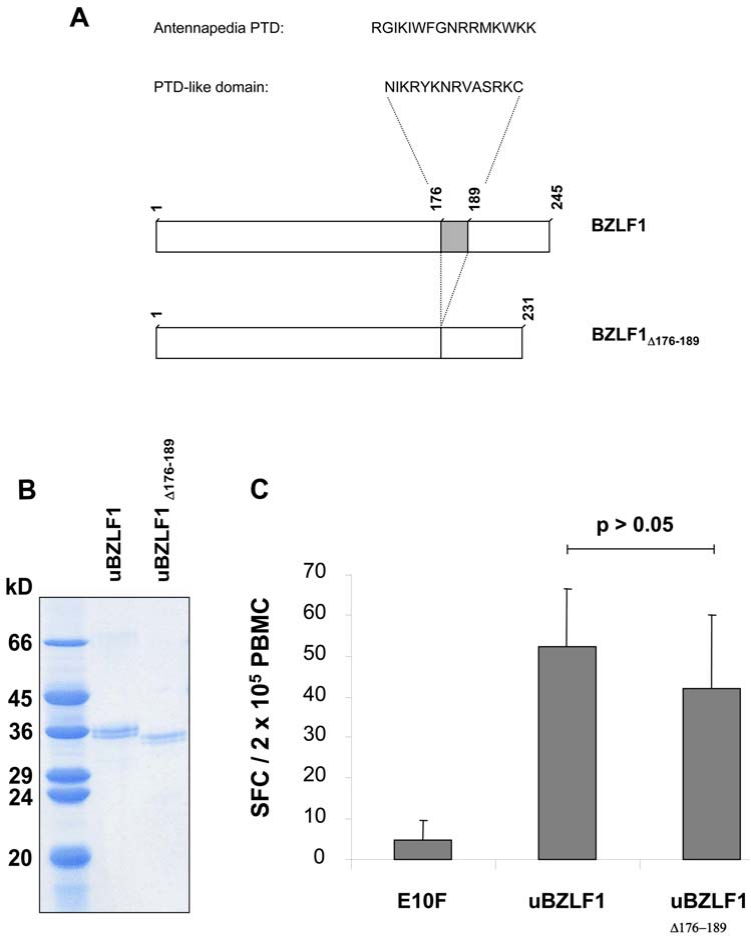
Deletion of a C-terminal arginine-rich domain within BZLF1 with homology to PTD does not abrogate the capacity of uBZLF1 to activate specific T cells. (A) A truncated BZLF1 protein, engineered by deletion of the arginine-rich domain (BZLF1Δ_176–189_) was expressed in *E. coli*, purified and treated with urea as described in materials and methods. (B) The purity of BZLF1 and BZLF1Δ_176–189_ was assessed by Coomassie staining. (C) PBMC of healthy EBV-seropositive donors were stimulated for 20 h with 10 µg/ml uBZLF1, BZLF1Δ_176–189_ or the HIV control peptide E10F and the frequencies of IFN-γ producing cells were assayed by ELISpot. The data show mean spot forming cell (SFC) values±standard deviation (s.d.) of 5 replicate stimulations and are a representative of results performed with PBMC of 3 different individuals.

During the last decade short arginine-rich protein-transduction domains (PTD) have been described to mediate polypeptide translocation across cell membranes. Sequence alignments revealed the presence of an arginine-rich domain within the C-terminal region of BZLF1 exhibiting similarities to a known PTD domain of the Antennapedia (Antp) homeoprotein (Fig. 3A). To examine the role of this PTD-like domain in the translocation of uBZLF1 into APC, we expressed a deletion mutant (BZLF1_Δ176–189_), lacking the putative PTD in *E. coli* (Fig. 3B). We stimulated PBMC of HLA B8-positive EBV-seropositive healthy donors with either 10 µg/ml uBZLF1 or truncated uBZLF1_Δ176–189_ and determined the number of specifically restimulated, IFN-γ secreting T cells by ELISpot. PBMC stimulated with HIV Gag-derived peptide E10F served as negative control. In these experiments, uBZLF1 and uBZLF1_Δ176–189_ showed no significant differences in their capacity to reactivate BZLF1-specific T cells (Fig. 3C), indicating that the predicted PTD within BZLF1 plays no significant role in urea-mediated protein translocation into the endogenous processing pathway of APC.

### uBZLF1 enters APC via a cross-presentation pathway

**Figure 4:**
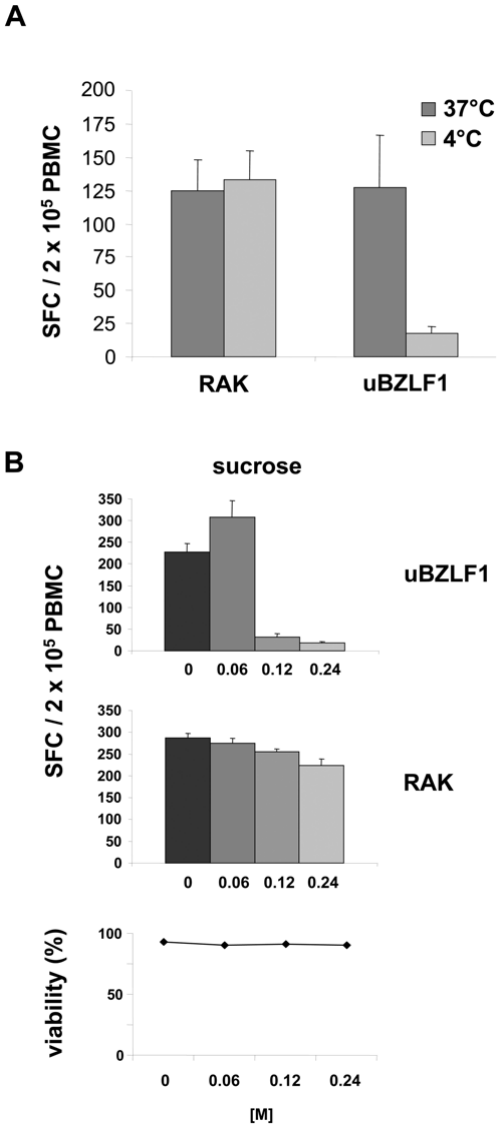
Uptake of uBZLF1 occurs in temperature-dependent manner by clathrin-mediated endocytosis. (A) This uptake and cross-presentation of uBZLF1-derived epitopes is inhibited at 4°C. PBMC were pulsed for 2 h at 4°C or 37°C with uBZLF1 or RAK peptide. The number of BZLF1-specific lymphocytes was determined by ELISpot. (B) Cross-presentation of uBZLF1 is blocked by sucrose, an inhibitor of clathrin-mediated endocytosis. Donor PBMC were stimulated with either uBZLF1 or the RAK peptide in presence of the indicated concentrations sucrose and the frequency of IFN-γ secreting cells was analyzed. Sucrose did not exhibit cytotoxic effects on PBMC at the used concentrations, as shown by PI-staining. All ELISpot data show mean SFC values+s.d. of 5 replicate stimulations and are representative of results performed with PBMC of 2 different individuals.

We used specific inhibitors of the HLA class I processing and presentation pathway to evaluate the molecular mechanisms underlying the urea-mediated uptake, processing and presentation of BZLF1. In order to study the energy dependency of uBZLF1 uptake into APC, PBMC were pre-incubated for 45 min at 4°C or 37°C and subsequently pulsed at the same temperature with uBZLF1 or the RAK peptide for an additional two hours. After removal of excessive antigen by washing, cells were incubated for an additional 20 h at 37°C and the numbers of IFN-γ producing T cells were determined by ELISpot. Pre-incubation and pulsing of cells with uBZLF1 at 4°C resulted in significantly reduced numbers of IFN-γ producing T cells compared to PBMC pulsed at 37°C, indicating a temperature sensitive and thus energy-dependent uptake of uBZLF1. In contrast, the efficiency of CTL reactivation by the RAK peptide was independent of the pre-incubation and pulsing temperature (Fig. 4A).

To further analyze the internalization process of uBZLF1, PBMC were pre-treated and stimulated for 20 h with uBZLF1 or RAK peptide in the presence of sucrose, an inhibitor of clathrin-mediated endocytosis. Specific activation of CTL by uBZLF1-pulsed PBMC was substantially abrogated in a dose dependent manner by sucrose, while CTL activation by the RAK peptide was hardly affected (Fig. 4B). Herein, sucrose did not exhibit cytotoxic effects on PBMC at the used concentrations, as shown by propidium iodide (PI) staining. These results indicate an uptake of uBZLF1 by clathrin-mediated endocytosis.

**Figure 5:**
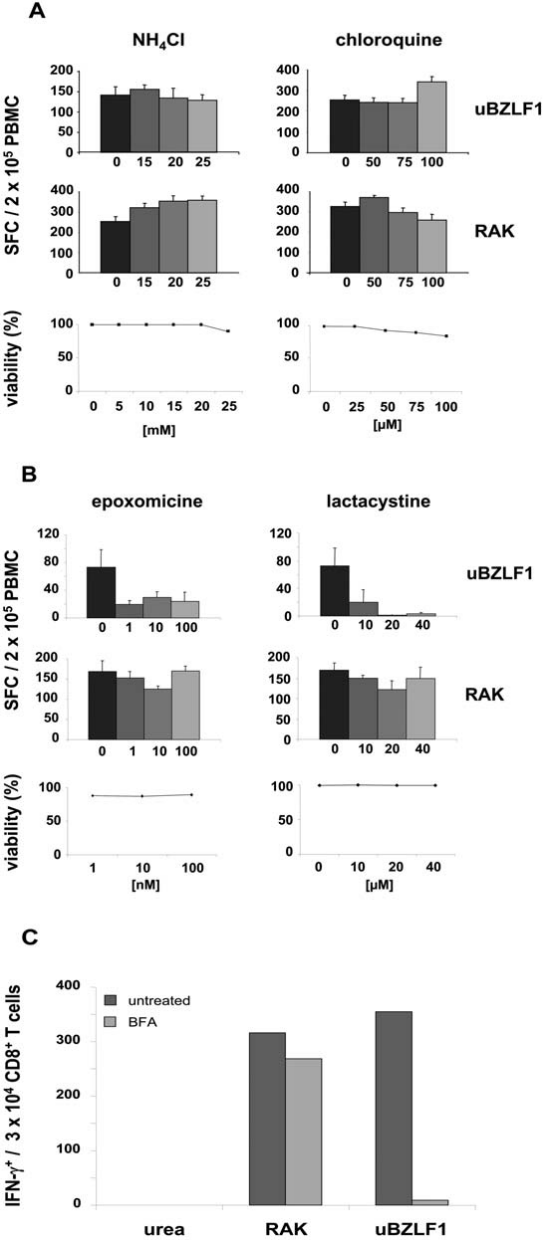
uBZLF1 is processed by a cross-presentation pathway. Cross-presentation of uBZLF1 is not influenced by (A) inhibition of endolysosomal acidification using chloroquine or NH_4_Cl, (B) but is significantly abrogated in presence of the proteosomal inhibitors epoxomycine and lactacystine. The ELISpot data show mean SFC values+s.d. of 5 replicate stimulations and are representative of results performed with PBMC of 3 different individuals. None of the applied drugs exhibited cytotoxic effects on PBMC at the used concentrations, as shown by PI-staining. (C) BFA strongly inhibits cross-presentation of uBZLF1. Whole blood was stimulated with uBZLF, RAK-peptide or, as control, 0.04 M urea in presence or absence of BFA. IFN-γ production was analyzed by intracellular staining using flow cytometry. The data show the number of specifically reactivated IFN-γ positive CD8^+^ T cells and are representative of experiments performed with 3 different donors.

To examine the role of proteolytic processes within endolysosomal compartments in the processing of uBZLF1 for epitope presentation on HLA class I molecules, we pre-treated PBMC for 45 min with two inhibitors of lysosomal acidification, NH_4_Cl or chloroquine [25],[26], and subsequently stimulated cells for an additional 20 h with uBZLF1 or RAK peptide in presence of the indicated inhibitors. Treatment of PBMC of HLA B8-positive, EBV-seropositive donors with both inhibitors did not significantly influence the capacity of uBZLF1 and the RAK peptide to specifically stimulate T cells for IFN-γ production, strongly indicating that lysosomal protein degradation plays no significant role in the generation of HLA class I presentable epitopes (Fig. 5A).

To analyze the involvement of the proteasome in the degradation of uBZLF1, PBMC of EBV-seropositive donors were cultured and stimulated with uBZLF1 or the RAK peptide for 20 h in the presence of epoxomicine or lactacystine, both inhibitors of proteasomal proteases. These substances caused substantial impairment of uBZLF1-mediated CTL activation, with lactacystine showing a stronger inhibitory effect than epoxomycine (Fig. 5B). In contrast, both compounds showed no significant inhibition of T cell reactivation upon stimulation with the RAK peptide. These results demonstrate that processing of uBZLF1 requires proteolytic activities from the proteasome complex.

Subsequently, we determined the sensitivity of HLA class I presentation of uBZLF1-derived epitopes to brefeldin A (BFA), an inhibitor of anterograde endoplasmic reticulum-golgi transport and hence an inhibitor of the endogenous HLA class I presentation pathway. Therefore, we pre-incubated whole blood of EBV-seropositive, HLA B8-positive donors with BFA for 45 min prior to stimulation with uBZLF1, the RAK control peptide or a 0.04 M urea solution for additional 7 h and analyzed the frequency of specifically reactivated CTL by intracellular IFN-γ staining. BFA significantly abrogated uBZLF1-induced reactivation of specific T cells, whereas activation of CTL by the RAK peptide was not significantly influenced (Fig. 5C), implicating that newly synthesized HLA class I molecules are required for an efficient presentation of uBZLF1-derived peptides. Taken together, these observations strongly suggest that the capacity of uBZLF1 to restimulate specific CTL relies on the uptake and processing of the antigen via a cross-presentation pathway.

### Dendritic cells, monocytes and B cells are capable of cross-presenting uBZLF1

**Figure 6:**
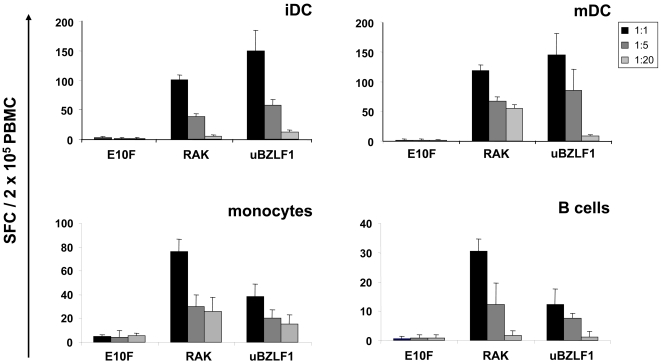
iDC, mDC, monocytes and B cells are capable to process uBZLF1 for epitope-presentation to CTL. iDC, mDC, monocytes, and B cells from EBV-seropositive, HLA B8-positive individuals were incubated for 2 h with 10 µg/ml uBZLF1, RAK or the E10F control peptide, washed and co-cultured with autologous PBMC at the indicated ratios. Numbers of positive cells were determined by ELISpot. The data show mean SFC values+s.d. of 5 replicate stimulations and are representative for 3 independent experiments with different donors.

We analyzed different subpopulations of APC for their ability to cross-present epitopes from uBZLF1. Therefore monocyte-derived immature (iDC) and mature DC (mDC), as well as monocytes and B cells were generated from PBMC of HLA B8-positive individuals, pulsed with uBZLF1, washed extensively and co-cultured with autologous PBMC. Activation of T cells was assessed by IFN-γ ELISpot. APC pulsed either with RAK or E10F peptides served as positive and negative controls. Dendritic cells pulsed with uBZLF1 showed a higher ability to restimulate antigen-specific T cells than monocytes and B cells. DC loaded with uBZLF1 were more efficient in the restimulation of IFN-γ producing T cells than DC incubated with the RAK peptide, while the opposite effect was observed for monocytes and B cells (Fig. 6). These results indicate that various APC subpopulations may account for cross-presentation of uBZLF1 and subsequent reactivation of BZLF1-specific T cells in PBMC or whole blood cultures.

## Discussion

In the present study we exemplified with the EBV immediate early protein BZLF1 a novel technology for the translocation of exogenous, soluble proteins into the HLA class I processing and presentation pathway of APC, which relies on the solubilisation of proteins with high molar urea and incubation of cells with these antigens in presence of low molar urea. In this study, we used BZLF1 as model antigen, because it includes an immunodominant HLA B8-restricted CTL epitope in addition to T helper cells epitopes [24],[27]. In addition, numbers of BZLF1-specific CTL are known to remain readily detectable over a long period of time, whereas specific T helper (Th) cell frequencies rapidly decline and persist at a low level following an acute infection [28].

*In vitro* stimulation of lymphocytes from HLA B8-positive, EBV-seropositive individuals with urea-solubilized BZLF1 revealed its strong capacity to restimulate BZLF1-specific CTL in addition to low numbers of IFN-γ producing Th cells. In some of the tested individuals, uBZLF1 specifically reactivated higher numbers of CTL than the RAK peptide. This finding can be explained by the activation of CTL with specificity for additional CTL epitopes within BZLF1 (manuscript in preparation).

Similar to uBZLF1 urea-formulated recombinant CMV pp65 protein revealed an enhanced capacity to reactivate pp65-specific CD8^+^ T cells when compared to dialyzed, urea-free pp65 supporting the applicability of the urea-based strategy for proteins other than BZLF1.

The transfer of uBZLF1 into the HLA class I processing and presentation pathway was shown to be mediated by urea as BZLF1 purified from urea by extensive dialysis lost its capacity to reactivate BZLF1-specific CTL.

Unlike native EBV EBNA1 protein, which has been shown to enter the endogenous presentation pathway of dendritic cells and EBV lymphoblastoid cell lines (LCL) upon elongated exposure of 12 hours [29],[30], urea-free BZLF1 did not activate CD8^+^ T cells even after 20 hours of incubation. The molecular mechanisms underlying urea-mediated translocation of BZLF1 are still unclear. One possible explanation would be, that urea-denaturated proteins may translocate more efficiently than correctly folded proteins.

Dowdy and co-workers showed that complete denaturation in 8 M urea and subsequent removal of urea by ionic exchange chromatography or dialysis strongly enhances the translocation efficiency of some proteins linked to the PTD of the HIV Tat protein [31]. However, we showed that removal of urea from urea-denatured BZLF1 drastically abrogated its capacity to enter the endogenous processing pathway of APC.

Urea in the applied concentration revealed neither cytotoxic nor immune activating properties in cultured blood cells and immature dendritic cells, ruling out the possibility of unspecific activation of APC or T lymphocytes. Thus, effectiveness of urea formulation considerably differs from that of uric acid, which promotes the capacity of co-applied proteins to prime CTL responses by the stimulation of dendritic cell maturation [32],[33].

BZLF1 contains a stretch of basic amino acid residues bearing resemblance to the PTD of Antp [34]. Several reports have demonstrated that PTD are able to transfer exogenous proteins across biological membranes into the cytosol of cells [21],[35],[36]. Initially discovered in the Tat protein of HIV [21] and in the Drosophila melanogaster Antp protein [37], these domains share clusters of basic amino acid residues, most commonly arginines, which mediate protein translocation into target cells. After binding of PTD to heparan sulfate on cell surfaces, APC internalize these proteins efficiently by macropinocytosis [38],[39]. Yet, deletion of this domain from BZLF1 had no significant influence on the urea-mediated transfer of BZLF1 into the HLA class I processing pathway, indicating that the arginine-rich domain of BZLF1 does not play a causal role in the cross-presentation of uBZLF1.

Uptake of uBZLF1 into APC was proceeded by a temperature sensitive process, suggesting that the antigen is internalized by endocytosis, phagocytosis or macropinocytosis [40],[41] rather than direct penetration across the lipid bilayer. This assumption is further supported by the finding that cross-presentation of uBZLF1 is dose dependently abrogated in presence of sucrose, strongly suggesting that internalization of uBZLF1 occurs by clathrin-mediated endocytosis [42]. However, it is likely that uBZLF1 is rescued from endolysosomal degradation by release into the cytoplasm or to less acidic compartments, as cross-presentation of uBZLF1 is neither sensitive to chloroquine nor NH_4_Cl, two drugs known to prevent endosomal acidification. As an alternative pathway uBZLF1 might be transferred to the endoplasmic reticulum by retrograde transport pathways as previously described for the B fragment of shiga toxin [43]. The cellular transport pathway that facilitates the escape of uBZLF1 from destruction by lysosomal proteases remains unclear, since no well-defined mechanisms have been described for the transfer of endocytic structures to the cytosol.

However, the impairment of uBZLF-induced CTL activation by epoxomicine and lactacystine, two inhibitors of proteasomal proteases clearly demonstrates the translocation of uBZLF1 to the cytosol and the essential role of the proteasome in the generation of uBZLF1-derived CTL epitopes.

The recognition of epitopes by CTL requires the interaction of a peptide-loaded HLA class I complex with specific T cell receptors. Our study shows that processed peptides are loaded onto new synthesized HLA class I molecules, as re-stimulation of BZLF1-specific CTL is completely abrogated in presence of BFA, a known inhibitor of protein secretion. Combined with our inhibition experiments of the proteasome this finding excludes the possibility of peptide loading on recycling HLA class I molecules in endolysosomal compartments [13]. Furthermore, these results rule out extracellular processing and loading of BZLF1 peptides on HLA molecules, as previously shown for HLA class II-restricted epitopes in immature dendritic cells [44].

Cross-presentation is a feature especially described for DC [11] and monocytes [4]. The ability of B cells to mediate cross-presentation is discussed controversial [45]–[47]. Our results showed that uBZLF1 is mainly cross-presented by iDC and mDC, while monocytes and B cells displayed reduced capacity to cross-present uBZLF1. The fact that both iDC and mDC are equally capable of cross-presenting uBZLF1 seems to be surprising, regarding their different capacity to capture antigens by phagocytosis and pinocytosis [48]. Yet, this finding supports our previous hypothesis of the cellular uptake of uBZLF1 by endocytosis, as this internalization route is not affected by the maturation state of DC [49].

Taken together, the data presented in this study reveal that the urea adjuvation technology constitutes a simple and useful tool for the concurrent *ex vivo* re-activation of antigen-specific CTL and Th cells. Compared to peptides the use of full length proteins provides the advantage of independence of HLA restriction. To circumvent this limitation large numbers of overlapping peptides would have to be synthesized. Another advantage of full length proteins is a natural processing of antigenic peptides and even spliced epitopes could be generated by this means [50]. Therefore this technology could improve the development of T cell diagnostics in a variety of viral, cancer and autoimmune diseases and may facilitate a novel strategy for the expansion of protein-specific CD8^+^ T cells for therapeutic applications.

## Materials and Methods

### Synthetic peptides and recombinant proteins

The synthetic peptide RAK, representing a HLA B8-restricted CTL epitope within EBV BZLF1 (aa190–197) [24] and the control peptide E10F, covering a murine CTL epitope within the p24 capsid region of HIV-1_(BH10)_ Gag (aa291–300) [51] were purchased from Synpep and reconstituted at 10 mg/ml in dimethylsulfoxide. Recombinant CMV pp65 protein was purchased from Miltenyi Biotec, Bergisch Gladbach, Germany.

### Expression and purification of BZLF1 and BZLF1_Δ176–189_

The open reading frame encoding the cDNA (+1-735, 245 amino acids) of EBV BZLF1 was inserted into the pET5a plasmid (Novagen) using the EcoRI and BamHI sites. For generation of a plasmid encoding BZLF1_Δ176–189_, nucleotides 525–567 were removed by deletion PCR. The resulting plasmids were called pET5a-Z and pET5a-Z_Δ176–189_, respectively. For the production of BZLF1 and BZLF1_Δ176–189_, *E. coli* BL21-DE3/CP-RIL were transformed with the pET5c-Z or pET5a-Z_Δ176–189_ vectors, grown at 26°C to an O.D._600_ of 0.8 and induced overnight with 1 mM IPTG. Full-length BZLF1 was expressed at 26°C for 20 h, truncated BZLF1 variant was expressed for 3 h at 37°C. Cells were harvested by centrifugation (10,000 g, 10 min) and stored at −80°C until further processing. Full-length BZLF1 was solubilized with urea from inclusion bodies, whereas truncated BZLF1 mutant was expressed in a soluble form and urea-denatured during purification process.

Frozen cells were thawed and resuspended in buffer (BZLF1: 20 mM Bis-Tris propane (BTP), 5 mM EDTA, 100 mM NaCl, pH 7.0; BZLF1_Δ176–189_: 20 mM Tris-HCl, 1 mM EDTA, 50 mM NaCl, pH 7.5). Cells were lysed by two passages through the high pressure Basic-Z system (IUL Instruments) at 1,500 bar. To avoid DNA contamination crude extracts were incubated with 2 U/ml Benzonase (20 min, RT). Subsequently, cell lysates were cleared by centrifugation (30,000 g, 10 min, 4°C).

For purification of full-length BZLF1, insoluble BZLF1 precipitate was washed (2 M urea, 20 mM BTP, 50 mM NaCl, pH 7.0) and then solubilized overnight in buffer containing 4 M urea (100 mM Tris, 4 M urea, 2 mM DTT, pH 7.5, overnight). After precipitation (30,000 g, 20 min), supernatant was further purified from contaminating proteins via acid precipitation. Therefore, pH was adjusted to 3.5 by addition of 1N HCl and further incubated for 30 min. Precipitated proteins were removed by centrifugation and BZLF1 containing supernatant was dialyzed (4 M urea, 20 mM citric acid, pH 3.5) and then loaded on a cation exchange column (Source S (GE Healthcare Life Sciences)). BZLF1 containing fractions eluted at ∼500 mM NaCl. BZLF1 containing fractions were pooled, dialyzed against (4 M urea, 20 mM Tris-HCl, 2 mM DTT, pH 7.5) and further purified by anion exchange chromatography (Source Q (GE Healthcare Life Sciences)). Pooled BZLF1 containing fraction (75–150 mM NaCl) was finally purified by gel filtration (Superdex 75 (GE Healthcare Life Sciences)).

For purification of truncated BZLF1_Δ176–189_, BZLF1_Δ176–189_ was first precipitated by dialysis against 4 M urea, 20 mM formic acid, 50 mM NaCl, 1 mM EDTA, pH 4.0. After centrifugation (17,000 g, 15 min) BZLF1_Δ176–189_ was resolubilized by dialysis (6 M urea, 50 mM formic acid, 50 mM NaCl, pH 4.0). Soluble denatured BZLF1_Δ176–189_ containing fraction was loaded on a cation exchange column (Source Q (GE Healthcare Life Sciences)). Pooled BZLF1_Δ176–189_ fraction (300–600 mM NaCl) was neutralized (4 M urea, 20 mM Tris-HCl, 2 mM DTT, pH 7.5) and further purified by anion exchange chromatography (Source Q (GE Healthcare Life Sciences)). Finally, BZLF1_Δ176–189_ fraction (∼100 mM NaCl) was purified by gel filtration (Superdex 75 (GE Healthcare Life Sciences)) chromatography for removal of further protein contamination from the expression host.

Resulting full-length BZLF1 and truncated BZLF1_Δ176–189_ revealed a purity of >95%. BZLF1 or BZLF1_Δ176–189_ were adjusted to a final concentration of 1 mg/ml in 4 M urea with 2 mM DTE prior to use in stimulation experiments.

### Cell preparation

Whole blood was obtained from healthy EBV seropositive volunteers with known HLA compositions. The study was approved by the Institutional Ethics Committee. PBMC were prepared from heparinized blood by standard Ficoll-Paque (PAN) density centrifugation, washed twice in PBS, resuspended in RPMI-1640 medium containing 10% (vol/vol) human AB-serum and 1% (vol/vol) antibiotics (streptomycin 10 mg/ml; penicillin 10 mg/ml) and adjusted to 1×10^7^ cells/ml. B cells and monocytes were purified from peripheral blood by MACS Sort magnetic beads through positive selection using anti-CD19 and anti-CD14 antibodies in MACS LS separation columns (Miltenyi Biotech) according to the manufacturer's protocol. Cell purity was assessed by flow cytometry using anti-CD20 (clone M5E2) and anti-CD14 (clone 2H7) (BD) antibodies. Production and phenotypical characterization of immature and mature dendritic cells was performed as previously described [52]. The purity of the APC populations was beyond 98%.

### Detection of IFN-γ secreting T cells by ELISpot

IFN-γ ELISpot assays were performed as described previously [53]. Briefly, 2×10^5^ PBMC were plated in 5 replicates into 96-well ELISpot plates (MAHA N45, Millipore) pre-coated with anti-IFN-γ mAb 1D-1K (5 µg/ml) (MabTech) and stimulated overnight at 37°C. After removal of the cells, the plates were developed with the IFN-γ specific biotinylated antibody 7-B6-1 (1 µg/ml) (MabTech) and streptavidine-alkaline phosphatase. Visualization of spots was performed with nitroblue tetrazolium substrate solution (Roche). IFN-γ SFC were counted with a Bioreader 2000 (ByoSys).

### Flow cytometry analysis of intracellular cytokines

For intracellular cytokine staining, heparinized whole blood from healthy EBV positive donors was incubated for 7 h at 37°C, the last 4 h in presence of 10 µg/ml BFA (Sigma), with either 10 µg/ml of the indicated antigens or 0.04 M urea-buffer as control. Anti-CD49d and anti-CD28 monoclonal antibodies (BD) were added for co-stimulation according to the manufacturer's protocol. The following reagents were used for flow cytometric analysis, unless otherwise noted: fluorescein isothiocyanate (FITC)-conjugated anti-CD8 (clone B9.11), phycoerythrin-texas-red (ECD)-conjugated anti-CD3 (clone UCHT1), cychrome™ (Pc5)-labelled anti-CD4 (clone 13B8.2) and a phycoerythrin (PE)-conjugated anti-IFN-γ antibody (clone 45.15) (all Beckman Coulter). Intracellular and surface markers were stained following fixation and permeabilisation of cells. Stained cells were run on a FACS Epics XL MCL flow cytometer (Beckman Coulter). Live-gating of lymphocytes and CD3^+^ events was performed during acquisition. Up to 2×10^6^ events were acquired for each analysis. Results were reported as percentage of the gated population producing IFN-γ.

### Antigen presentation assays and inhibitory compounds

Pure APC populations were pulsed with 10 µg/ml uBZLF1 or RAK peptide for 2 h at 37°C, washed twice and co-cultured with autologous PBMC for 20 h. For the analysis of the presentation pathway of uBZLF1, the following inhibitory compounds were used at indicated concentrations: sucrose, epoxomicin, lactacystin, NH_4_Cl, chloroquine (all from Sigma). The working concentration of BFA (Sigma) was 10 µg/ml. In antigen processing and presentation assays, PBMC or whole blood samples were pre-treated with the respective inhibitory compounds 45 min prior to pulsing with the indicated antigens. The compounds remained in the culture throughout the incubation. The number of IFN-γ producing cells was assessed by ELISpot after stimulation of PBMC for 20 h or by FACS analysis following stimulation of whole blood for 7 h, respectively. Cell viability was analyzed by staining with propidium iodide (BD), according to the manufacturer's protocol.

### Statistical analysis

Statstical analysis was evaluated using the Mann-Whitney U-test in SPSS for independent samples. A value of p<0.05 was considered significant.

### Accession numbers

The accession number of the BZLF1 protein used in this study is YP_401673.

## Supporting Information

Figure S1 Numbers and ratios of specifically reactivated CD8^+^ T cells and Th cells differ in uBZLF1, BZLF1 and RAK peptide stimulated blood cells of individual donors. Heparinized blood samples of four to six different HLA B8, EBV-seropositive donors were pulsed with 10 µg/ml uBZLF1, dialyzed BZLF1 or RAK peptide and the percentage of IFN-γ positive Th cells and CD8^+^ T cells was assessed by flow cytometry. In these experiments we observed substantial differences in the total numbers and ratio of specifically reactivated CD8^+^ T cells upon stimulation with uBZLF1 and RAK. The removal of urea from uBZLF1 by extensive dialysis results in an almost complete loss of its capacity to restimulate CD8^+^ T cell (donor 1 to 4). Furthermore uBZLF1 stimulation of whole blood activated only limited (donor 1, 2) to undetectable (donors 3 to 6) numbers of Th cells for IFN-γ production. NT: not tested.
(0.64 MB TIF)

Figure S2 (u)pp65 (4 M urea) but not urea-free pp65 reactivates specific CD8^+^ T lymphocytes as determined by intracellular IFN-γ staining. In contrast, removal of urea from upp65 had no effect on its ability to specifically restimulate CD4^+^ T cells. Whole blood from a CMV-seropositive donor was stimulated with either 10 µg/ml of pp65, upp65 or for control with 0.04 M urea. Cells were incubated for 7 h at 37 C° with 10 µg/ml BFA added for the last 4 h. Shown is the percentage of CD3^+^CD8^+^ or CD3^+^CD4^+^ cells expressing IFN-γ from a representative stimulation of three independent experiments using different donors. Plots show log fluorescence intensity.
(1.42 MB TIF)

Figure S3 Urea does not induce secretion of cytokines from PBMC. PBMC were incubated with 0.04 M urea solution. Cells stimulated with medium alone, 100 ng/ml lipopolysaccharide (LPS) or 1 µg/ml phorbol 12-myristate 13-acetate (PMA)/Ionomycin served as negative and positive controls. After 24 h cell-free supernatants were collected and analyzed for depicted cytokines using a Luminex 100™ and the Human UltraSensitive Cytokine Ten-Plex Antibody Bead Kit. Values exceeding the detection limit are indicated by asterisks. The data represent mean values of two independent stimulations +s.d.
(1.43 MB TIF)

Figure S4 Urea does not activate dendritc cells. iDC were generated by cultivation of monocytes in the presence of 500 µg/ml GM-CSF and IL-4 for 5 days. iDC were stimulated for 48 hours with 0.04 M urea solution and anayzed for the expression of costimulatory molecules and HLA-DR by flow cytometry. Cells stimulated with medium alone, 100 ng/ml LPS or Ionuleit cocktail (10 ng/ml IL-1β, 10 ng/ml TNFα, 1000 U/ml IL-6, 1 µg/ml PGE_2_) served as negative and positive controls. The data shown are representative of 2 independent experiments with iDC of different donors.
(2.02 MB TIF)
