# Supplementary figures and images for: Urea-Mediated Cross-Presentation of Soluble Epstein-Barr Virus BZLF1 Protein
Source: PLoS Pathog. 2008 Nov 7;4(11):e1000198. doi: 10.1371/journal.ppat.1000198 (PMC2572144; doi:10.1371/journal.ppat.1000198)

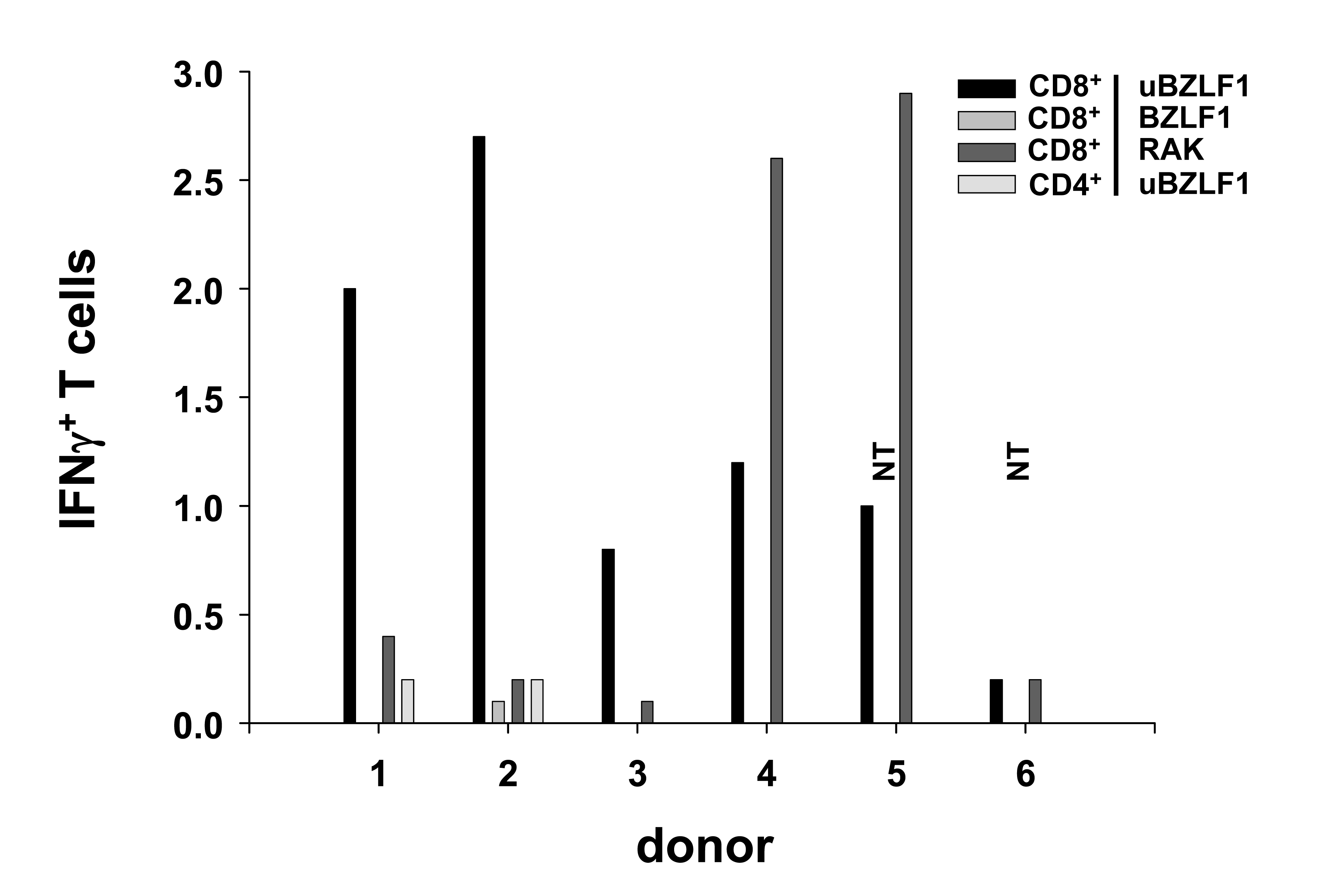

Supplement: Figure S1 — Numbers and ratios of specifically reactivated CD8+ T cells and Th cells differ in uBZLF1, BZLF1 and RAK peptide stimulated blood cells of individual donors. Heparinized blood samples of four to six different HLA B8, EBV-seropositive donors were pulsed with 10 µg/ml uBZLF1, dialyzed BZLF1 or RAK peptide and the percentage of IFN-γ positive Th cells and CD8+ T cells was assessed by flow cytometry. In these experiments we observed substantial differences in the total numbers and ratio of specifically reactivated CD8+ T cells upon stimulation with uBZLF1 and RAK. The removal of urea from uBZLF1 by extensive dialysis results in an almost complete loss of its capacity to restimulate CD8+ T cell (donor 1 to 4). Furthermore uBZLF1 stimulation of whole blood activated only limited (donor 1, 2) to undetectable (donors 3 to 6) numbers of Th cells for IFN-γ production. NT: not tested. (0.64 MB TIF) [file ppat.1000198.s001.tif]

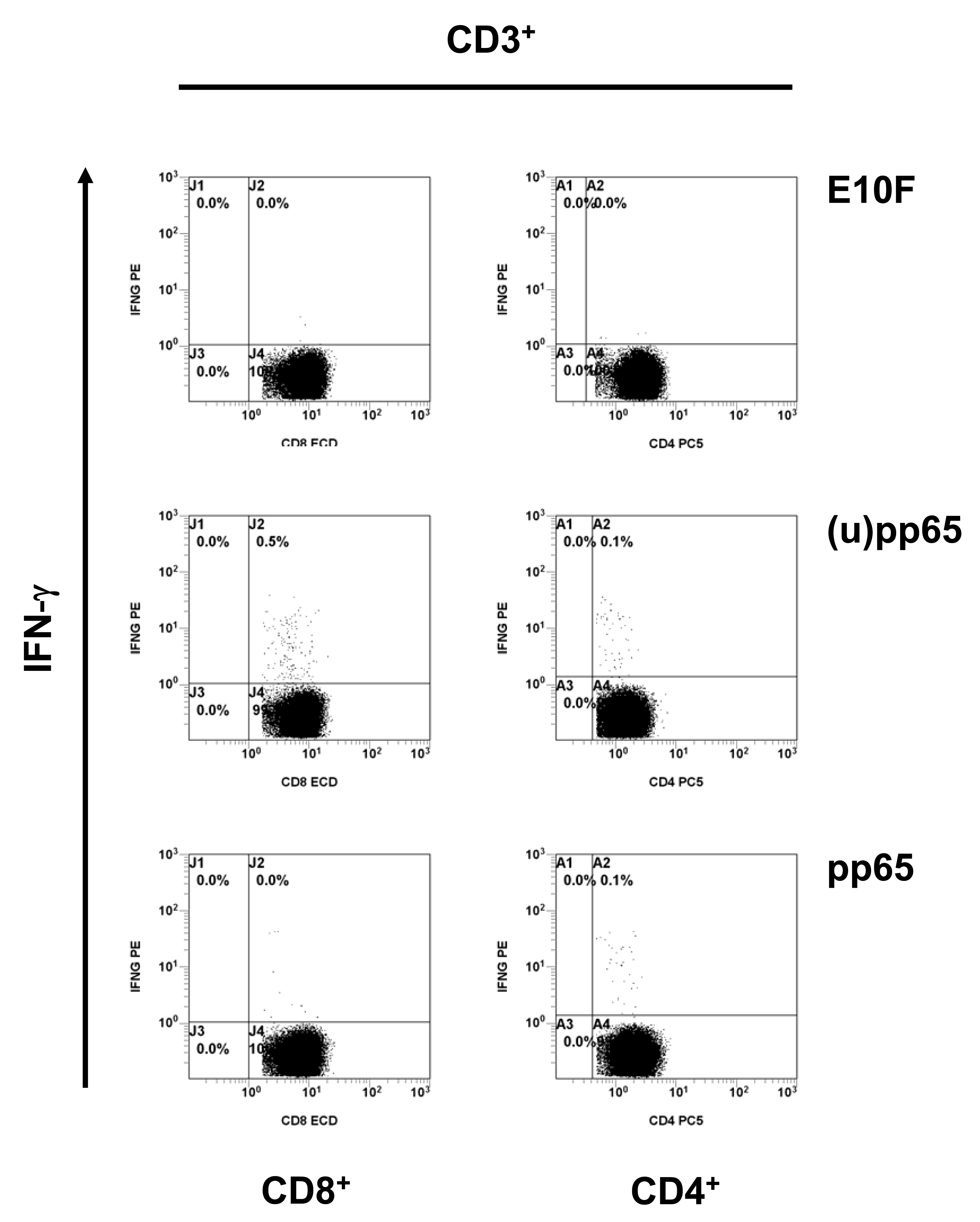

Supplement: Figure S2 — (u)pp65 (4 M urea) but not urea-free pp65 reactivates specific CD8+ T lymphocytes as determined by intracellular IFN-γ staining. In contrast, removal of urea from upp65 had no effect on its ability to specifically restimulate CD4+ T cells. Whole blood from a CMV-seropositive donor was stimulated with either 10 µg/ml of pp65, upp65 or for control with 0.04 M urea. Cells were incubated for 7 h at 37 C° with 10 µg/ml BFA added for the last 4 h. Shown is the percentage of CD3+CD8+ or CD3+CD4+ cells expressing IFN-γ from a representative stimulation of three independent experiments using different donors. Plots show log fluorescence intensity. (1.42 MB TIF) [file ppat.1000198.s002.tif]

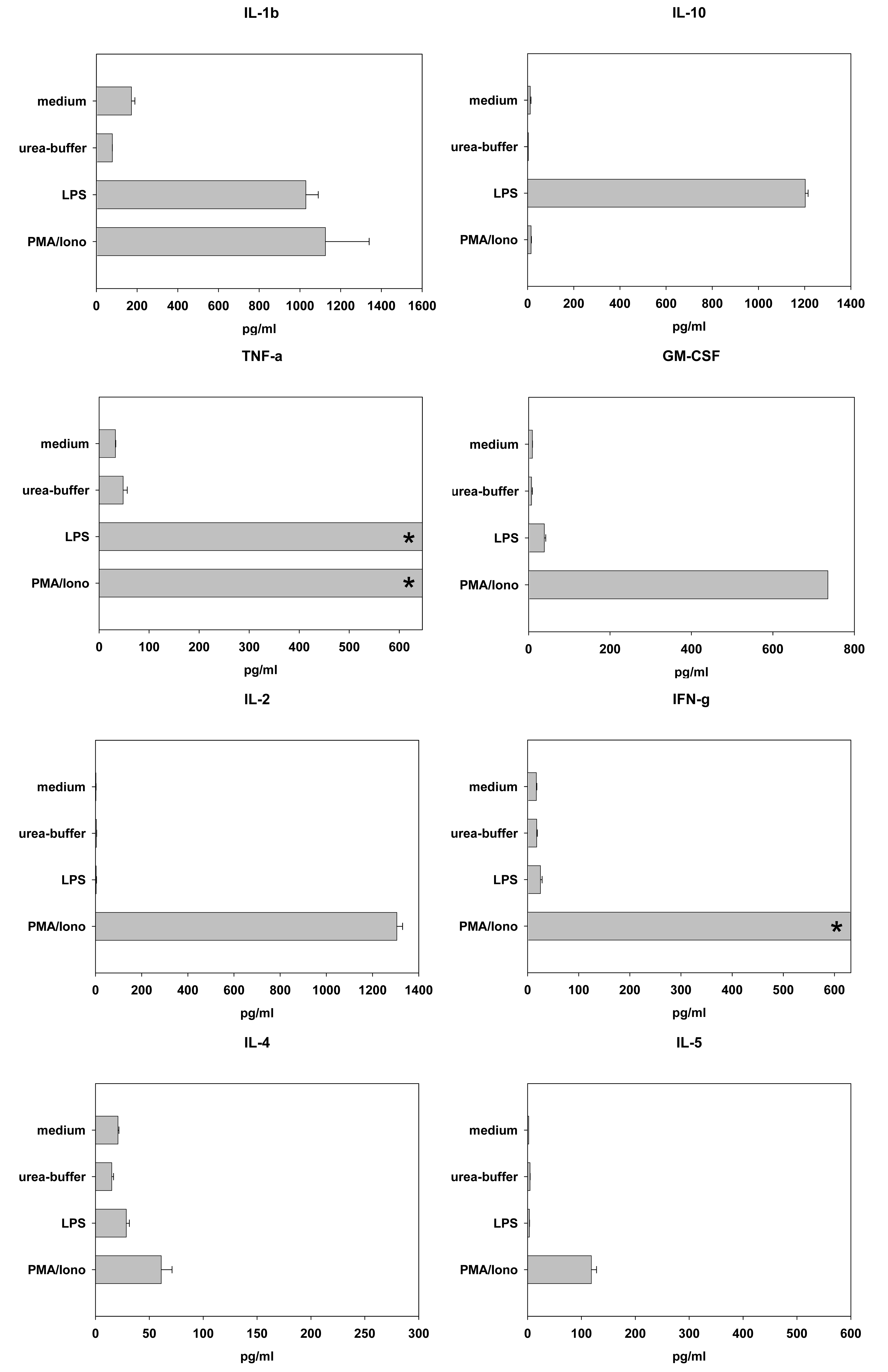

Supplement: Figure S3 — Urea does not induce secretion of cytokines from PBMC. PBMC were incubated with 0.04 M urea solution. Cells stimulated with medium alone, 100 ng/ml lipopolysaccharide (LPS) or 1 µg/ml phorbol 12-myristate 13-acetate (PMA)/Ionomycin served as negative and positive controls. After 24 h cell-free supernatants were collected and analyzed for depicted cytokines using a Luminex 100™ and the Human UltraSensitive Cytokine Ten-Plex Antibody Bead Kit. Values exceeding the detection limit are indicated by asterisks. The data represent mean values of two independent stimulations +s.d. (1.43 MB TIF) [file ppat.1000198.s003.tif]

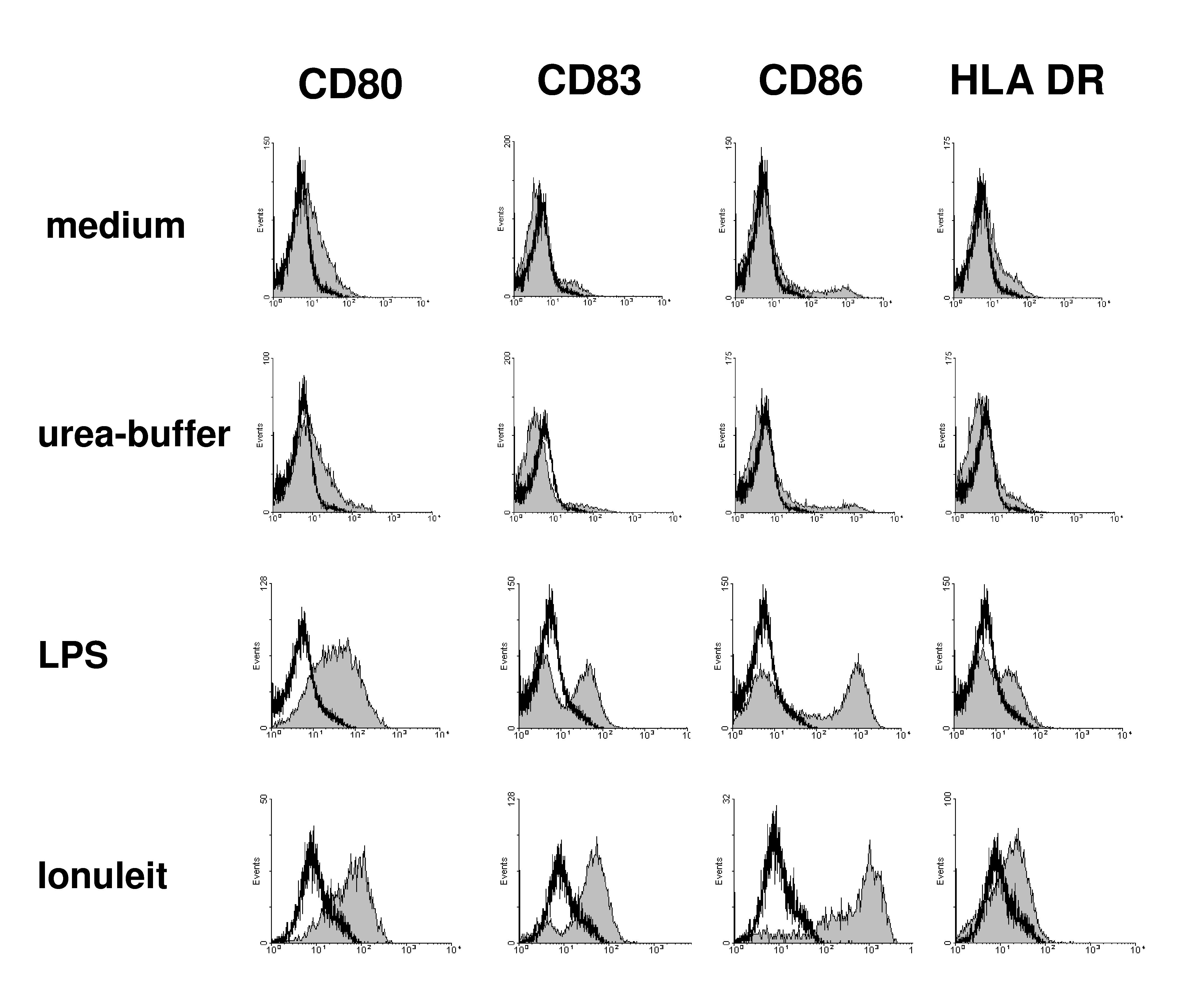

Supplement: Figure S4 — Urea does not activate dendritc cells. iDC were generated by cultivation of monocytes in the presence of 500 µg/ml GM-CSF and IL-4 for 5 days. iDC were stimulated for 48 hours with 0.04 M urea solution and anayzed for the expression of costimulatory molecules and HLA-DR by flow cytometry. Cells stimulated with medium alone, 100 ng/ml LPS or Ionuleit cocktail (10 ng/ml IL-1β, 10 ng/ml TNFα, 1000 U/ml IL-6, 1 µg/ml PGE2) served as negative and positive controls. The data shown are representative of 2 independent experiments with iDC of different donors. (2.02 MB TIF) [file ppat.1000198.s004.tif]
